# Selective Benefit of Adjuvant Chemotherapy in Stage II dMMR Colon Cancer with High-Risk Features or Poorly Differentiated Histology: A Retrospective Study

**DOI:** 10.3390/cancers18040629

**Published:** 2026-02-14

**Authors:** Yonglin Huang, Yuye Gao, Yingjie Li, Xingyu Xie, Junpeng Pei, Yunfeng Yao, Tiancheng Zhan, Nan Chen, Jiahua Leng, Lin Wang, Jun Zhao, Aiwen Wu

**Affiliations:** State Key Laboratory of Holistic Integrative Management of Gastrointestinal Cancers, Beijing Key Laboratory of Carcinogenesis and Translational Research, Unit III, Gastrointestinal Cancer Center, Peking University Cancer Hospital & Institute, Beijing 100142, China; 2311110742@stu.pku.edu.cn (Y.H.); 2411110707@stu.pku.edu.cn (Y.G.);

**Keywords:** colonic neoplasms, adjuvant therapy, chemotherapy, survival, mismatch repair

## Abstract

Background: The prognostic value of conventional high-risk factors and the benefits of adjuvant chemotherapy (ACT) in stage II colon cancer with deficient mismatch repair (dMMR) remain controversial. The function of ACT in stage II dMMR colon cancer and survival results were assessed in this research. Methods: 273 patients with stage II dMMR colon cancer who had curative resection between August 2010 and October 2023 underwent a retrospective analysis. Clinicopathologic variables, postoperative treatment strategies, and survival endpoints were systematically assessed. Independent prognostic factors were identified using a multivariable Cox proportional hazards regression model. For subgroup analyses, a propensity score–matched (PSM) approach was used to minimize intergroup imbalances. Overall survival (OS) and disease-free survival (DFS) were evaluated using the Kaplan–Meier approach. Results: 177 (64.8%) patients had at least one high-risk factor. With a median follow-up of 62.6 months, the estimated 5-year OS and DFS rates were 94.7% and 89.8%. Age ≥ 65 years and examination of fewer than 12 lymph nodes were independently associated with OS. For DFS, age ≥ 65 years, LNs < 12, and receipt of ACT were identified as independent prognostic factors. According to subgroup analyses, ACT was linked to better OS and DFS in patients with high-risk features or poorly differentiated histology. Results were similar after propensity score matching. Conclusion: Traditional high-risk features also exert prognostic impact on this population. ACT appeared to be associated with improved survival in selected high-risk patients, particularly those with poorly differentiated histology.

## Simple Summary

Deficient mismatch repair (dMMR) stage II colon cancer generally has a favorable prognosis, yet the prognostic value of conventional high-risk features and the benefit of adjuvant chemotherapy (ACT) remain controversial. We retrospectively analyzed 273 patients with stage II dMMR colon cancer who underwent curative resection at Peking University Cancer Hospital between August 2010 and October 2023. Overall, 31.9% received ACT (predominantly CAPOX or capecitabine). With a median follow-up of 62.6 months, 5-year overall survival (OS) and disease-free survival (DFS) were 94.7% and 89.8%. Age ≥ 65 years and examination of fewer than 12 lymph nodes were independent adverse factors for OS, while age ≥ 65 years, fewer than 12 lymph nodes, and ACT status were independently associated with DFS. Subgroup and propensity score–matched analyses suggested that ACT was associated with improved survival in selected patients, particularly those with high-risk features and those with poorly differentiated histology, whereas no clear benefit was observed in low-risk or well/moderately differentiated subgroups.

## 1. Introduction

Stage II colon cancer is a heterogeneous disease that generally exhibits favorable long-term outcomes following curative resection. However, approximately 10–20% of patients experience disease recurrence, underscoring the persistent challenge of identifying individuals who may benefit from adjuvant chemotherapy (ACT) [1]. Major clinical guidelines recognize several high-risk characteristics, such as pT4 disease, poor differentiation, lymphovascular or perineural invasion, bowel obstruction or perforation, and insufficient lymph node evaluation, as important factors when considering adjuvant chemotherapy for stage II colon cancer [2]. Evidence regarding the survival effect of ACT in patients with dMMR or MSI-H tumors remains inconsistent.

Approximately 15% of stage II colon cancers exhibit a dMMR/MSI-H phenotype, which is commonly characterized by right-sided tumor predominance, mucinous or signet-ring cell histology, as well as a generally favorable prognosis [1,3]. Poorly differentiated histology is generally linked to a worse prognosis, but whether this adverse effect also applies to patients with dMMR tumors remains unclear. Multiple pooled analyses have demonstrated that patients with dMMR status derive little or no benefit from fluoropyrimidine-based adjuvant therapy and may even experience detrimental effects [4,5]. In spite of this, a number of retrospective series and subgroup analyses from pivotal trials, such as MOSAIC and NSABP C-07, suggest that certain subgroups may still achieve survival improvements from oxaliplatin-based regimens, especially those with T4 stage, poorly differentiated histology, or inadequate lymph node sampling [6]. These contradictory results underscore the necessity for current real-world evidence to accurately determine the prognostic value of high-risk factors and the potential benefits of ACT in patients with dMMR/MSI-H status.

Accordingly, we conducted a retrospective cohort study to examine the prognostic impact of conventional high-risk clinicopathological features and to explore the association between ACT and survival outcomes in patients with stage II dMMR colon cancer, especially among high-risk patients. By providing updated survival data in this population, our study offers contemporary real-world evidence to inform individualized postoperative management strategies.

## 2. Methods

### 2.1. Patients and Treatment

**Figure 1:**
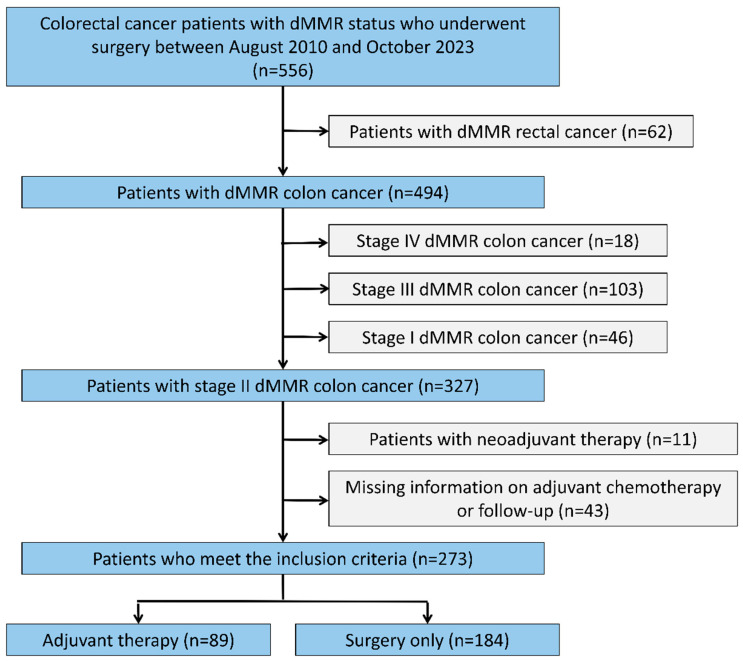
Flow diagram of patients with stage II dMMR colon cancer who underwent curative resection. dMMR: deficient mismatch repair.

A retrospective review was performed on 556 patients with gastrointestinal malignancies and confirmed dMMR status who received curative surgical resection at Peking University Cancer Hospital (PKUCH) from August 2010 to October 2023. The eligible patients met the following criteria: (i) pathologically verified primary colon cancer (CC), (ii) pathologically confirmed dMMR/MSI status, and (iii) Stage II disease was defined according to the eighth edition of the American Joint Committee on Cancer (AJCC) staging system. In the final analysis, a total of 273 patients who met the inclusion criteria were included (Figure 1). From the medical records, clinical data, including patient demographics, medical history, ACT regimens, surgical procedures, and pathological characteristics, were extracted. This study protocol was approved by the Institutional Review Board of Peking University Cancer Hospital (approval No. 2022YJZ39). Given its retrospective nature, informed consent was waived.

### 2.2. Adjuvant Chemotherapy

All adjuvant chemotherapy regimens were commenced within 3–6 weeks following surgery. Patients receiving capecitabine monotherapy or CAPOX (Capecitabine + Oxaliplatin regimen) underwent treatment every 3 weeks for a total duration of 24 weeks (eight cycles). Patients receiving FOLFOX (Leucovorin + 5-Fluorouracil + Oxaliplatin regimen) were treated every 2 weeks for 24 weeks (12 cycles). Dose adjustments were made according to predetermined clinical guidelines based on observed toxicities. For advanced patients or individuals with a poor Eastern Cooperative Oncology Group performance (ECOG ≥ 3), a strategy of observation without ACT was adopted.

### 2.3. Definition of High-Risk Stage II Colon Cancer

Patients with stage II colon cancer were classified as high risk when at least one adverse clinicopathological feature was present. These features included poor histologic differentiation (including undifferentiated or poorly differentiated adenocarcinoma, mucinous adenocarcinoma, or signet-ring cell carcinoma), lymphovascular (LVI) or perineural invasion (PNI), pT4 stage, preoperative bowel obstruction or perforation, positive resection margins, or inadequate lymph node examination (<12 lymph nodes).

### 2.4. Determination of MMR Status

The MMR status was assessed using immunohistochemical (IHC) staining to evaluate the expression of MMR proteins, including MLH1, MSH2, MSH6, and PMS2, which are critical markers for mismatch repair deficiency. To ensure diagnostic accuracy and consistency, IHC staining was assessed by experienced pathologists independently. Tumors were defined as deficient mismatch repair (dMMR) when loss of expression of one or more mismatch repair (MMR) proteins was observed, whereas tumors with intact expression of all MMR proteins were classified as proficient mismatch repair (pMMR).

### 2.5. Statistical Analysis

Descriptive statistics were summarized as proportions and medians. Categorical variables were compared using the chi-square test or Fisher’s exact test as appropriate. Disease-free survival (DFS) was defined as the time from surgery to the first occurrence of tumor recurrence, metastasis, or death from any cause, whereas overall survival (OS) was defined as the interval from surgery to death from any cause. Patients who were alive without evidence of recurrence or metastasis at the last follow-up were censored. OS and DFS were estimated using the Kaplan–Meier method, and survival curves were compared between subgroups stratified by adjuvant chemotherapy status using the log-rank test. All subgroup analyses were considered exploratory; therefore, *p*-values were not adjusted for multiple testing. A multivariable Cox proportional hazards regression model was applied using the enter method to assess survival outcomes and identify independent prognostic factors, incorporating variables that were statistically significant in univariable analyses as well as those considered clinically relevant. To assess potential selection bias associated with the missing data, baseline characteristics were compared between patients with complete datasets and those with incomplete datasets. Hazard ratios (HRs) with corresponding 95% confidence intervals (CIs) were estimated for each covariate, and a two-sided *p* value < 0.05 was considered statistically significant. Statistical analyses were performed using SPSS (version 22.0), GraphPad Prism (version 9.2.0), and R software (version 4.2.0).

### 2.6. Sensitivity Analysis

To further control for selection bias in this retrospective cohort, a sensitivity analysis based on propensity score matching was conducted. Using the MatchIt package, propensity scores were calculated through a logistic regression model incorporating key baseline variables, including age, sex, tumor location, and ECOG performance status. A 1:1 nearest-neighbor matching strategy was applied to pair ACT recipients with comparable patients in the surgery-only arm, enforcing a strict caliper threshold of 0.02. Following the establishment of this balanced cohort, the primary survival analyses were repeated to validate the robustness of our findings.

## 3. Results

### 3.1. Characteristics of Patients

Table 1 summarizes the patients in clinicopathological characteristics. Between 2010 and 2023, a total of 273 consecutive patients with dMMR stage II colon cancer were identified, of whom 177 (64.8%) presented with at least one high-risk feature. The median patient age was 58 years (range: 18–88 years), and 57.5% were male, while 42.5% were female. The most frequent high-risk factors were poorly differentiated histology (48.0%), followed by pT4 tumor stage (13.2%), preoperative complications such as bowel obstruction or perforation (11.7%), LVI + (11.4%), PNI + (9.9%), and LNs < 12 (4.4%). In total, 68.1% of the 184 patients were treated with surgery alone, while 89 patients (31.9%) received ACT followed by surgery. Among those, 70.8% received the CAPEOX regimen, 27.0% received capecitabine alone, and 2.2% received the FOLFOX regimen. In the high-risk group, 72 patients (40.7%) received ACT, compared with 49 patients (36.8%) in the poorly differentiated histology group. Missing data occurred more often in earlier treatment periods, but baseline characteristics were comparable between patients with complete and incomplete datasets (Table S1).

### 3.2. Actual Long-Term Survival and Independent Factors for OS and DFS

The median follow-up duration was 62.6 months. The 3- and 5-year OS rates were 96.7% and 94.7%, respectively, while the corresponding DFS rates were 93.7% and 89.8%. In univariate analysis, age ≥65 years (HR 6.03; 95% CI, 2.02–17.99; *p* = 0.001) and LNs < 12 (HR 8.73; 95% CI, 2.73–27.88; *p* < 0.001) were significantly associated with poorer OS. In a similar manner, age ≥65 years (HR 4.79; 95% CI, 1.98–11.57; *p* < 0.001), LNs < 12 (HR 6.89; 95% CI, 2.52–18.85; *p* < 0.001), and LVI (HR 3.01; 95% CI, 1.18–7.70; *p* = 0.022) were link to a poorer DFS. After adjustment in a multivariable Cox proportional hazards model, age ≥65 years (HR 5.58; 95% CI, 1.32–23.65; *p* = 0.020) and LNs < 12 (HR 5.20; 95% CI, 1.15–23.45; *p* = 0.032) remained independent prognostic factors for OS. Regarding DFS, age ≥65 years (HR 4.19; 95% CI, 1.44–12.17; *p* = 0.009), LNs < 12 (HR 5.63; 95% CI, 1.60–19.84; *p* = 0.007), and without ACT (HR 0.12; 95% CI, 0.01–0.92; *p* = 0.041) with a substantially increase risk (Table 2).

### 3.3. Survival According to Stage II dMMR, High-Risk, Poorly Differentiated Histology Groups, and Adjuvant Therapy

**Figure 2:**
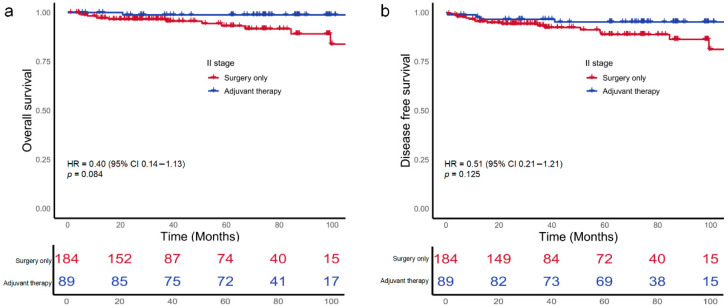
Kaplan–Meier survival curves for overall-survival (**a**) and disease-free survival (**b**) of patients with stage II dMMR colon cancers stratified by treatment.

**Figure 3:**
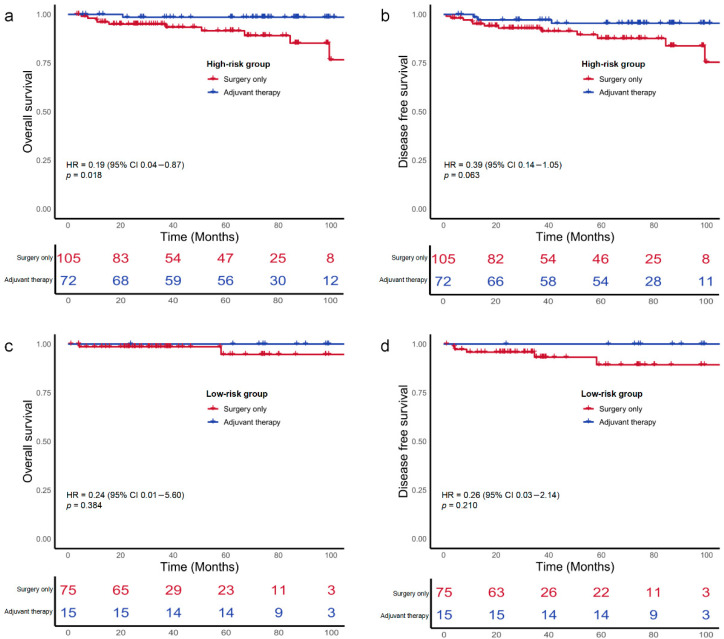
Kaplan–Meier survival curves for overall-survival (OS) and disease-free survival (DFS) of patients with stage II dMMR colon cancers stratified by high-risk statuses and treatment. (**a**) OS in high-risk group. (**b**) DFS in high-risk group. (**c**) OS in low-risk group. (**d**) DFS in low-risk group.

**Figure 4:**
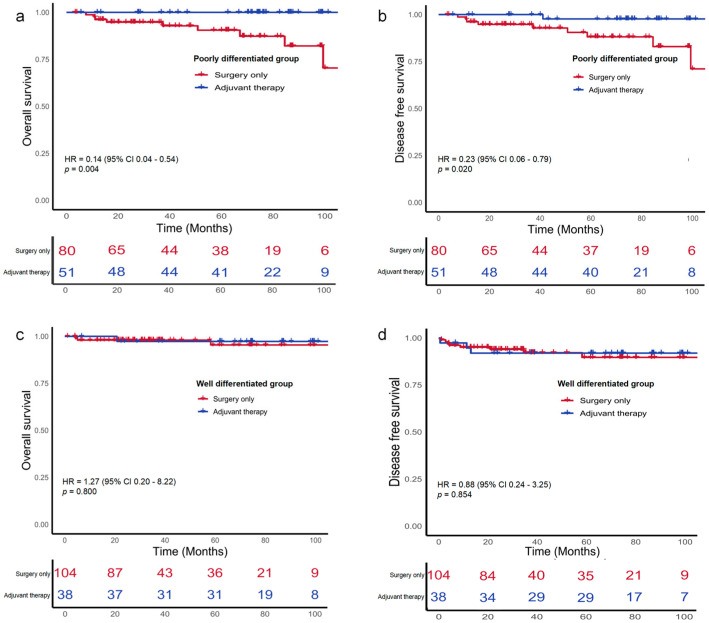
Kaplan–Meier survival curves for overall-survival (OS) and disease-free survival (DFS) of patients with stage II dMMR colon cancers stratified by histological differentiation and treatment. (**a**) OS in poorly differentiated group. (**b**) DFS in poorly differentiated group. (**c**) OS in well differentiated group. (**d**) DFS in well differentiated group.

Kaplan–Meier estimates showed that 5-year OS rates was 93.2% among the surgery-only group and 98.8% among the ACT group (HR 0.40; 95% CI, 0.14–1.13; *p* = 0.084; Figure 2a). The corresponding 5-year DFS rates did not differ significantly between the surgery-only and adjuvant chemotherapy groups (88.9% vs. 95.4%; HR, 0.51; 95% CI, 0.21–1.21; *p* = 0.125; Figure 2b). Among the 177 patients presenting with at least one high-risk feature, the 5-year OS and DFS were 98.6% and 95.6% in those receiving ACT, compared with 91.7% and 87.8% in those treated with surgery alone, although these differences did not reach statistical significance (Figure 3a,b). Furthermore, among the 131 patients with poorly differentiated histology, those receiving ACT demonstrated significantly better 5-year OS and DFS compared to those who underwent surgery alone (100% vs. 90.7%, (HR 0.14, 95% CI, 0.04–0.54, *p* = 0.004); 97.7% vs. 88.3%, (HR 0.23, 95% CI, 0.06–0.79, *p* = 0.020); Figure 4a,b).

By contrast, in low-risk or those with well or moderately differentiated histology subgroups, OS and DFS did not differ significantly between individuals managed with surgery alone and receiving ACT (Figure 3 and Figure 4).

### 3.4. Survival According to Other Variables, Subgroups, and Adjuvant Therapy Regimens

Notably, distinct patterns of ACT benefit were observed between anatomical and biological risk factors (Figure S1). No significant survival benefit from ACT was observed in subgroups defined by anatomical complexity, including pT4 stage (OS *p* = 0.497), preoperative complications (OS *p* = 0.628), and LNs < 12 (OS *p* = 0.663). In contrast, ACT significantly improved or showed a trend towards improved outcomes in patients with aggressive biological features, such as PNI (DFS *p* = 0.038), poorly differentiated histology (Figure 4), and LVI (OS *p* = 0.057). Because no patients had a positive surgical margin and ECOG performance status showed minimal variability, these variables were not included in the analysis.

In the comparison of ACT regimens, 24 patients received capecitabine monotherapy. Survival outcomes did not differ significantly between patients treated with capecitabine alone and those receiving oxaliplatin-based regimens, with respect to either OS or DFS (Figure S1).

### 3.5. Sensitivity Analysis

To rigorously address the potential selection bias and confounding factors (such as age imbalances) identified between the treatment groups, we performed a PSM analysis [7]. The baseline covariates, including age, gender, ECOG performance status, and tumor location, were well-balanced after matching, as demonstrated by the Love plots (Figure S2).

**Figure 5:**
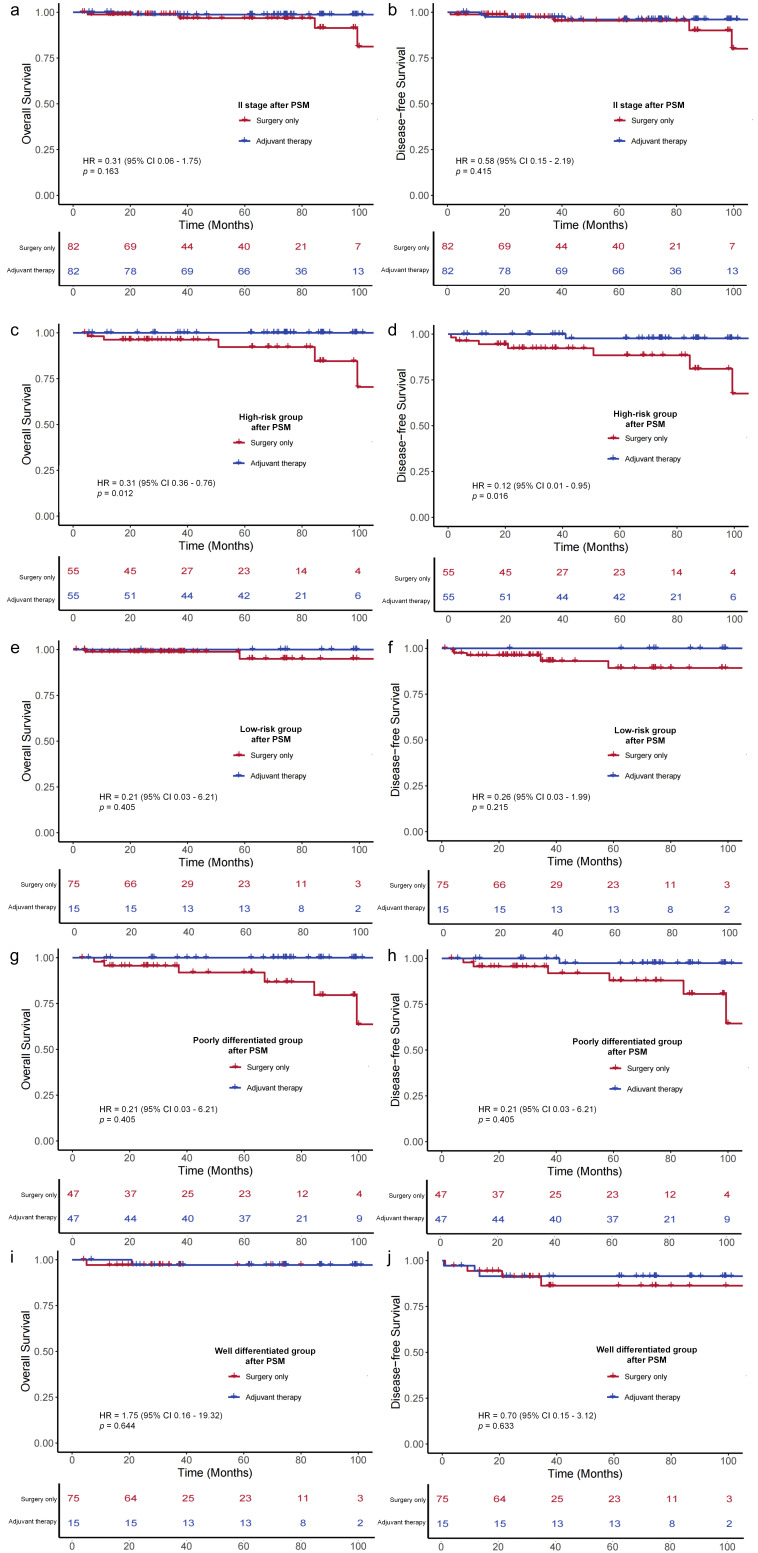
Kaplan–Meier survival curves for overall-survival (OS) and disease-free survival (DFS) of patients with stage II dMMR colon cancers after propensity score matching (PSM). (**a**) OS in patients with stage II dMMR colon cancers. (**b**) DFS in patients with stage II dMMR colon cancers. (**c**) OS in high-risk group. (**d**) DFS in high-risk group. (**e**) OS in low-risk group. (**f**) DFS in low-risk group. (**g**) OS in poorly differentiated group. (**h**) DFS in poorly differentiated group. (**i**) OS in well differentiated group. (**j**) DFS in well differentiated group.

In the propensity score–matched high-risk cohort, patients receiving ACT showed statistically superior outcomes in both OS (*p* = 0.012) and DFS (HR, 0.12; 95% CI, 0.01–0.95; *p* = 0.016) (Figure 5). Similarly, in the matched poorly differentiated histology subgroup, patients receiving ACT exhibited superior survival outcomes compared to the surgery-only group (OS: *p* = 0.003; DFS: HR 0.12, 95% CI 0.01–0.99, *p* = 0.018). Conversely, consistent with the primary analysis, no statistically significant survival benefit from ACT was observed in the matched low-risk or well differentiated subgroup. These findings from the balanced cohorts reinforce the conclusion that the survival benefit of ACT in stage II dMMR colon cancer is selective to patients with high-risk features or poorly differentiated histology.

## 4. Discussion

According to our research, conventional high-risk factors exert varying effects on survival outcomes among patients with stage II dMMR colon cancer. Specifically, age ≥65 years and LNs < 12, LVI, and receipt of ACT had a greater impact on OS and DFS compared to other factors. Among patients with high-risk features, ACT was associated with a significant survival benefit, particularly in those with poorly differentiated histology, where both 5-year OS and DFS improved with ACT.

The dMMR/MSI-H molecular phenotype in colon cancer exhibits distinct clinical and pathological characteristics, primarily observed in earlier stages, with reported frequencies of approximately 22% in stage II, 12% in stage III, and 3.5% in stage IV [8]. Data from our institution were concordant with these previously reported distributions. According to current clinical practice guidelines from the European Society for Medical Oncology (ESMO) and the National Comprehensive Cancer Network (NCCN), poorly differentiated histology, pT4 stage, bowel obstruction or perforation, LNs <  12, LVI, and PNI are recognized as high-risk factors for stage II colon cancer, for which ACT is recommended [2,9]. In the present study, LNs < 12 and LVI were significantly correlated with poorer prognosis. These results align with the reports by David et al., Hermanek et al., and Scott et al., who emphasized that adequate lymph node assessment is essential for accurate staging, postoperative management, and improved survival outcomes, recommending the harvest of a minimum of 12 lymph nodes [10,11]. Moreover, Al-Sukhni et al. and Zhong et al. reported that LVI and PNI are related to worse outcomes [12,13]. Collectively, these observations indicated that specific conventional high-risk factors may influence the prognosis of patients with stage II dMMR colon cancer.

The interaction between histologic differentiation and MMR status remains poorly understood, particularly in patients with stage II disease. Compared with pMMR/MSS tumors, dMMR/MSI-H groups exhibit distinct histological features showing a markedly higher incidence of mucinous histology, signet-ring cell histology, as well as poorly or undifferentiated morphology pathologically [3]. According to Kim et al., the proportion of poorly differentiated histologic subtypes may exceed 30% among patients with the dMMR/MSI-H group [14]. Multiple studies have reported that unfavorable histologic differentiation serves as an independent prognostic risk factor for local recurrence and distant metastasis, thereby adversely affecting OS, DFS [15,16,17,18]. Compared with surgery alone, ACT has been recommended to confer potential survival benefits in this subgroup. However, some studies suggest that the MMR status itself may serve as a predictive and protective biomarker associated with favorable outcomes following surgery alone [19]. Despite this, the NCCN guidelines do not currently list poorly differentiated histology as a high-risk feature in stage II dMMR colon cancer, discouraging the use of ACT regardless of additional high-risk factors being present [2].

One direct consequence of this uncertainty is the ongoing controversy regarding the use of ACT in this population. A retrospective study from France, along with subgroup analyses of the NSABP C-07 and MOSAIC trials, suggested that adjuvant chemotherapy may confer a DFS benefit in patients with stage II dMMR colon cancer [20,21]. Conversely, other studies have shown no significant survival advantage or even a potential detrimental effect on OS associated with ACT, including in high-risk or poorly differentiated subgroups [22,23]. These contradictory findings may primarily stem from methodological issues. First, many cohorts were enrolled in the 1990s, when MMR testing was in its early stages, and standardized detection protocols were not widely implemented until the early 2000s [24]. Additionally, advances in surgical techniques, particularly the introduction of complete mesocolic excision (CME) in 2009, have fundamentally redefined the concept of radical colectomy. This approach enables a more comprehensive regional lymph node retrieval while minimizing surgical trauma. Consequently, CME has substantially improved patient outcomes and is now regarded as the standard surgical procedure for resectable colon cancer [25,26]. Moreover, much of the available evidence derives from retrospective subgroup analyses involving relatively small numbers of dMMR cases, thereby limiting statistical power and the capacity to detect clinically meaningful differences.

Given these limitations, we conducted a retrospective analysis of prognostic outcomes in patients with stage II dMMR colon cancer over the past decade, intending to provide updated evidence regarding the potential benefits of ACT for patients presenting with high-risk features or poorly differentiated histology. Our findings indicated that patients with poorly differentiated histology experienced worse OS and DFS than those with well-differentiated tumors, whereas adjuvant chemotherapy was associated with a potential survival benefit. As a sensitivity analysis, propensity score matching was performed, yielding similar results.

Extensive efforts have been made to optimize adjuvant treatment strategies for stage II colon cancer, with initial investigations primarily focusing on fluoropyrimidine-based therapies [27]. In the MOSAIC trial, 899 patients with stage II colon cancer (regardless of MMR status) were randomly assigned to receive either 5-fluorouracil/leucovorin (FL) alone or FOLFOX (oxaliplatin plus FL) as adjuvant therapy. Among them, 569 patients were classified as high risk. The HR for OS between the FOLFOX and FL groups was 0.91 (95% CI, 0.61–1.36), suggesting no statistically significant difference in survival outcomes between the two regimens [28]. Furthermore, analysis of the ACCENT database demonstrated that adding oxaliplatin to FL offered only marginal benefit in high-risk groups, even among those presenting with multiple high-risk features [19]. A retrospective long-term follow-up study involving stage II and III disease, stratified by MMR status, demonstrated that patients with MSI-L or MSS tumors experienced more favorable outcomes after treatment with 5-fluorouracil (5-FU)-based regimens. In contrast, no statistically significant survival benefit was observed among patients in the MSI-H subgroup who received 5-FU–based ACT [5]. In a similar vein, a pooled retrospective analysis of adjuvant treatment trials by Sargent et al. suggested that adjuvant treatment with 5-fluorouracil (5-FU) may have been detrimental in patients with stage II dMMR colon cancer [29]. Given the limited efficacy of FL monotherapy, several studies have investigated the addition of oxaliplatin to adjuvant regimens. In a retrospective French study involving 149 patients with high-risk stage II dMMR colon cancer, 24 patients received FOLFOX, 116 underwent surgery alone, and 9 received FL. Compared with patients who underwent surgery alone, those receiving FOLFOX exhibited a trend toward improved disease-free survival (HR, 0.13; 95% CI, 0.02–1.05; *p* = 0.06) [20]. Other studies, including the NSABP C-07 analysis, also reported a potential benefit of adding oxaliplatin to FL, irrespective of MMR status [30]. Similarly, subgroup analyzes of the MOSAIC trial appeared to support a potential improvement in both DFS and OS with the FOLFOX regimen (DFS: HR, 0.48; 95% CI, 0.21–1.12; *p* = 0.08; OS: HR, 0.41; 95% CI, 0.16–1.07; *p* = 0.69). In a separate cohort of 262 consecutive patients with stage II dMMR colon cancer, oxaliplatin-based adjuvant chemotherapy did not show a survival advantage; 3-year disease-free survival was 92.6% in the surgery-only group and 88.1% in the ACT group. (HR 1.64; 95% CI, 0.67–4.02; *p* = 0.280) [21].

Recent advances in managing resectable dMMR colon cancer are the introduction of immunotherapy and circulating tumor DNA testing. The ATOMIC trial, an international multicenter phase III randomized study, compared adjuvant mFOLFOX6 (control arm) with mFOLFOX6 plus atezolizumab (experimental arm) in patients with stage III dMMR colon cancer. The experimental arm demonstrated a 3-year disease-free survival (DFS) rate of 86.4% (95% CI, 81.8–89.9), indicating an absolute improvement of 10% compared to the control arm (76.6%; 95% CI, 71.3–81.0). The disparity in DFS was significant both statistically and clinically [27]. Given the substantial benefits observed in stage III disease, further well-designed prospective randomized controlled trials are warranted to determine whether similar effects can be achieved in stage II disease [31]. Moreover, circulating tumor DNA (ctDNA)–guided minimal residual disease (MRD) assessment has recently emerged as a promising tool for developing individualized adjuvant treatment strategies. In the DYNAMIC trial, a ctDNA-guided approach significantly reduced the use of ACT (15% vs. 28%) without compromising long-term outcomes. The 5-year OS was comparable between the ctDNA-guided group and standard management groups (93.8% vs. 93.3%) [32]. Likewise, evidence from the CIRCULATE-Japan study indicated that postoperative circulating tumor DNA positivity was strongly associated with poorer disease-free survival. Patients who were ctDNA-negative 4 weeks postoperatively experienced markedly improved DFS, highlighting the prognostic significance of early ctDNA clearance [33]. These advances in therapeutic and diagnostic modalities reinforce the need for clinicians to consider more individualized and precision-oriented postoperative strategies for patients in this subgroup.

This study has several inherent limitations associated with its single-institution, retrospective sample size, including the relatively small sample sizes in some subgroups analyzes (e.g., the poorly differentiated subgroup) that limited the confidence of the resulting estimates. Accordingly, these findings should be regarded as hypothesis-generating and warrant validation in larger, independent cohorts. An additional constraint of this study is the lack of BRAF V600E and KRAS mutation data. Due to the retrospective nature of our study (2010–2023), routine testing for these markers was not standard for stage II patients in earlier years. We acknowledge that an imbalance in BRAF V600E status could potentially confound our results. However, previous reports suggest that the strong protective effect of dMMR status in stage II disease may outweigh the negative prognostic impact of BRAF mutations [4]. Moreover, the study lacked detailed information on the chemotherapy duration and toxicity, which could potentially influence the observed survival outcomes. Finally, although our findings are not entirely novel, this study provides additional evidence and context to clarify areas where previous findings have been inconsistent.

Looking forward, while this study refines the selection of candidates for chemotherapy, traditional cytotoxic agents may not represent the ultimate solution for high-risk dMMR disease. Future precision medicine strategies represent more promising directions. Specifically, circulating tumor DNA (ctDNA) analysis has emerged as a powerful tool to detect minimal residual disease (MRD) and guide adjuvant therapy decisions, potentially superseding traditional clinicopathological risk factors. Moreover, in light of the high tumor mutational burden characteristic of dMMR tumors, immune checkpoint inhibitors (ICIs) have emerged as a transformative therapeutic strategy. Recent trials exploring neoadjuvant or adjuvant immunotherapy suggest that harnessing the immune system may offer superior efficacy compared to conventional chemotherapy, warranting further investigation in prospective trials.

## 5. Conclusions

Stage II dMMR colon cancer generally exhibits a favorable prognosis, and the selective use of adjuvant therapy may help further optimize treatment outcomes. Our study demonstrated that adjuvant therapy was associated with improved outcomes among patients with high-risk features or poorly differentiated histology. However, the balance between the magnitude of clinical benefit and potential treatment-related toxicities requires further evaluation in larger, prospective studies.

## Figures and Tables

**Table 1:** Clinicopathologic characteristics of stage II dMMR colon cancer patients stratified by high-risk status and histologic differentiation.

Characteristic	Total (N = 273)	High-Risk (N = 177)		*p*-Value	Poorly Differentiated (N = 131)		*p*-Value
		Surgery Only (N = 105, 59.3%)	Adjuvant Therapy (N = 72, 40.7%)		Surgery Only (N = 82, 62.6%)	Adjuvant Therapy (N = 49, 37.4%)	
Age(years)				0.069			0.091
≥65	88(32.2)	38(36.2)	16(22.2)		30(36.6)	11(22.4)	
<65	185(67.8)	67(63.8)	56(77.8)		52(63.4)	38(77.6)	
Sex				0.635			0.453
Male	157(57.5)	56(53.3)	41(56.9)		43(52.4)	29(59.2)	
Female	116(42.5)	49(46.7)	31(43.1)		39(47.6)	20(40.8)	
ECOG performance				0.650			1.000
0	267(97.8)	101(96.2)	71(98.6)		79(96.3)	48(98.0)	
1	6(2.2)	4(3.8)	1(1.4)		3(3.7)	1(2.0)	
Tumor location segment				0.944			0.928
Ascending colon	138(50.5)	52(49.5)	37(51.4)		43(52.4)	26(23.1)	
Transverse colon	39(14.3)	14(13.3)	7(9.7)		10(12.2)	6(12.2)	
Descending colon	36(13.2)	17(16.2)	11(15.3)		16(19.5)	7(14.3)	
Sigmoid colon	39(14.3)	14(13.3)	10(13.9)		7(8.5)	5(10.2)	
Multiple	22(8.1)	8(7.6)	7(9.7)		6(7.3)	5(10.2)	
Primary tumor location				0.931			0.852
Right side	168(61.5)	63(60.0)	44(61.1)		49(61.3)	33(64.7)	
Left side	82(30.0)	33(31.4)	21(29.2)		24(30.0)	13(25.5)	
Multiple	23(8.4)	9(8.6)	7(9.7)		7(8.8)	5(9.8)	
Preoperative CEA level ^a^				0.927			0.101
>5 ng/mL	79(29.4)	32(30.8)	22(31.4)		24(30.4)	16(32.7)	
≤5 ng/mL	190(70.6)	72(69.2)	48(68.6)		55(69.6)	33(67.3)	
Preoperative CA19-9 level ^b^				0.584			0.697
>37 U/mL	35(13.1)	12(11.7)	10(14.5)		8(10.3)	6(12.5)	
≤37 U/mL	232(86.9)	91(88.3)	59(85.5)		70(89.7)	42(87.5)	
Preoperative obstruction or Perforation				0.696			0.303
Yes	32(11.7)	18(17.1)	14(19.4)		7(8.5)	7(14.3)	
No	241(88.3)	87(82.9)	58(80.6)		75(91.5)	42(85.7)	
pT stage				0.892			0.710
T3	237(86.8)	84(80.0)	57(79.2)		69(84.1)	40(81.6)	
T4	36(13.2)	21(20.0)	15(20.8)		13(15.9)	9(18.4)	
No. of sampled LNs				0.126			0.257
≥12	261(95.6)	95(90.5)	70(97.2)		75(91.5)	48(98.0)	
<12	12(4.4)	10(9.5)	2(2.8)		7(8.5)	1(2.0)	
Margins				1.000			1.000
Positive	0	0	0		0	0	
Negative	273	105	72		82	49	
Lymphovascular invasion ^c^				0.159			0.267
Yes	31(11.4)	15(14.3)	16(22.5)		11(13.4)	10(20.8)	
No	241(88.3)	90(85.7)	55(77.5)		71(86.6)	38(79.2)	
Perineural invasion ^d^				0.013 *			0.560
Yes	27(9.9)	11(10.9)	16(25.8)		8(10.1)	6(14.0)	
No	226(82.8)	90(89.1)	46(74.2)		71(89.9)	37(86.0)	
Differentiation				0.425			1.000
Poorly differentiated	131(48.0)	80(76.2)	51(70.8)		82	49	
Moderately differentiated	142(52.0)	25(23.8)	21(29.2)		0	0	
Adjuvant chemotherapy				**-**			**-**
Yes	89(31.9)	0	72(40.7)		0	49(36.8)	
Capecitabine alone	24(27.0)	0	18(25.0)		0	13(26.5)	
CAPOX	63(70.8)	0	52(72.2)		0	34(69.4)	
FOLFOX	2(2.2)	0	2(2.8)		0	2(4.1)	
No	184(68.1)	105	0		82	0	
Local recurrence				1.000			1.000
Yes	5(1.8)	1(1.0)	1(1.4)		0	0	
No	268(98.2)	104(99.0)	71(98.6)		82	49	
Metastasis				1.000			1.000
Yes	6(2.2)	3(2.9)	2(2.8)		3(3.7)	1(2.0)	
No	267(97.8)	102(97.1)	70(97.2)		79(96.3)	48(98.0)	
Death				0.147			0.128
Yes	15(5.5)	10(9.5)	2(2.8)		9(11.0)	1(2.0)	
No	258(94.5)	95(90.5)	70(97.2)		73(89.0)	48(98.0)	

^a^: 4 cases had missing data for preoperative CEA information; ^b^: 6 cases had missing data for preoperative CA19-9 information; ^c^: 1 case had missing data for lymphovascular invasion information; ^d^: A total of 20 cases had missing data for perineural invasion information. dMMR: deficient mismatch repair, ECOG: Eastern Cooperative Oncology Group, CEA: carcinoembryonic antigen, CA19-9: carbohydrate antigen 199, pT stage: Pathological T stage, LNs: Lymph nodes, CAPOX: Capecitabine plus Oxaliplatin, FOLFOX: Leucovorin + Fluorouracil + Oxaliplatin. *: statistically significant *p*-values (*p* < 0.05).

**Table 2:** Univariate and multivariate analysis of OS and DFS in stage II dMMR patients.

Variable	Comparator vs. Reference	OS				DFS			
		Univariate Analysis		Multivariate Analysis		Univariate Analysis		Multivariate Analysis	
		HR (95% CI)	*p*-Value	HR (95% CI)	*p*-Value	HR (95% CI)	*p*-Value	HR (95% CI)	*p*-Value
Age(years)	≥65 vs. <65	6.03(2.02–17.99)	0.001 *	5.58(1.32–23.65)	0.020 *	4.79(1.98–11.57)	<0.001 *	4.19(1.44–12.17)	0.009 *
Gender	Male vs. Female	1.49(0.54–4.13)	0.443			1.32(0.57–3.05)	0.519		
CEA level	>5 ng/mL vs. ≤5 ng/mL	2.21(0.77–6.32)	0.139			1.75(0.72–4.30)	0.220		
CA19-9 level	>37 U/mL vs. ≤37 U/mL	1.11(0.24–5.19)	0.893			1.12(0.31–4.00)	0.868		
Preoperative complication	Yes vs. No	2.11(0.59–7.59)	0.252			1.24(0.37–4.21)	0.731		
Primary tumor location	Right vs. left vs. multiple	0.51(0.11–2.43)	0.673			0.57(0.16–2.00)	0.646		
Differentiation	Poorly vs. Moderately	2.12(0.72–6.21)	0.172			1.00(0.43–2.32)	0.997		
pT category	pT4 vs. pT3	2.42(0.76–7.65)	0.133			1.46(0.49–4.34)	0.495		
No. of sampled LNs	<12 vs. ≥12	8.73(2.73–27.88)	<0.001 *	5.20(1.15–23.45)	0.032 *	6.89(2.52–18.85)	<0.001 *	5.63(1.60–19.84)	0.007 *
Lymphovascular invasion	Yes vs. No	2.78(0.88–8.75)	0.080			3.01(1.18–7.70)	0.022 *	2.57(0.65–10.11)	0.177
Perineural invasion	Yes vs. No	1.36(0.30–6.21)	0.695			1.42(0.41–4.91)	0.583		
Adjuvant chemotherapy	Yes vs. No	0.36(0.10–1.29)	0.117			0.49(0.18–1.32)	0.158	0.12(0.01–0.92)	0.041 *

dMMR: deficient mismatch repair, CEA: carcinoembryonic antigen, CA19-9: carbohydrate antigen 199, pT stage: Pathological T stage, LNs: Lymph nodes. HR: hazard ratio; CI: confidence interval, OS: overall survival, DFS: disease-free survival. *: statistically significant *p*-values (*p* < 0.05).

## Data Availability

The original anonymous datasets are available from the corresponding authors on reasonable request.

## Supplementary Materials

The following supporting information can be downloaded at: https://www.mdpi.com/article/10.3390/cancers18040629/s1, Supplementary Table S1. Comparison of characteristics between patients with missing CEA, CA19-9, lymphovascular and perineural invasion data and the complete data group. Supplement Figure S1. Kaplan–Meier survival curves for overall-survival (OS) and disease-free survival (DFS) of patients with stage II dMMR colon cancers stratified by other variables and adjuvant therapy regimens. (a) OS in elderly patients group. (b) DFS in elderly patients group. (c) OS in fewer than 12 examined lymph nodes (LNs < 12) group. (d) DFS in LNs < 12 group. (e) OS in lymphovascular invasion positive (LVI+) group. (f) DFS in LVI+ group. (g) OS in perineural invasion positive (PNI+) group. (h) DFS in PNI+ group. (i) OS in preoperative complication group. (j) DFS in preoperative complication group. (k) OS in pT4 stage group. (l) DFS in pT4 stage group. (m) OS in XELOX or FOLFOX regimen group and Capecitabine alone group. (n) DFS in XELOX or FOLFOX regimen group and Capecitabine alone group. (o) OS in CEA > 5 ng/mL group. (p) DFS in CEA > 5 ng/mL group. (q) OS in CA19-9 > 37 U/mL group. (r) DFS in CA19-9 > 37 U/mL group. Supplement Figure S2. Covariate balance before and after adjustment in the: (a) total cohort; (b) high-risk group; (c) low-risk group; (d) poorly differentiated group; (e) well differentiated group.

## Abbreviations

The following abbreviations are used in this manuscript:

ACT	Adjuvant chemotherapy
CI	Confidence interval
CRC	Colorectal cancer
DFS	Disease-free survival
dMMR	Deficient mismatch repair
HR	Hazard ratio
LNs	Lymph nodes
MSI	Microsatellite instability
OS	Overall survival
