# Supplementary material for: Selective Benefit of Adjuvant Chemotherapy in Stage II dMMR Colon Cancer with High-Risk Features or Poorly Differentiated Histology: A Retrospective Study
Source: Cancers (Basel). 2026 Feb 14;18(4):629. doi: 10.3390/cancers18040629 (PMC12939670; doi:10.3390/cancers18040629)
Supplement: Supplementary file 1 [file cancers-18-00629-s001.zip › cancers-4095584-supplementary.pdf]

Supplementary materials

Supplementary Table S1 Comparison of characteristics between patients with missing CEA, CA19-9, lymphovascular and perineural invasion data and the complete data group

Characteristic	Incomplete dataset N=25	Complete dataset N=248	*p*-Value
Age(years)			0.355
≥65	19(76.0)	166(66.9)	
＜65	6(24.0)	82(33.1)	
Sex			0.491
Male	16(64.0)	141(56.9)	
Female	9(36.0)	107(43.1)	
ECOG performance			1.000
0	25	242(97.6)	
1	0	6(2.4)	
Primary tumor location			0.281
Right side	17(68.0)	151(60.9)	
Left side	8(32.0)	74(29.8)	
Multiple	0	23(9.3)	
Preoperative obstruction or Perforation			1.000
Yes	3(12.0)	29(11.7)	
No	22(88.0)	219(88.3)	
pT stage			0.755
T3	21(84.0)	216(87.1)	
T4	4(16.0)	32(12.9)	
No. of sampled LNs			1.000
≥12	24(96.0)	237(95.6)	
＜12	1(4.0)	11(4.4)	
Margins			1.000
Positive	0	0	
Negative	25	248	
Differentiation			0.673
Poorly differentiated	12(48.0)	130(52.4)	
Moderately differentiated	13(52.0)	118(47.6)	
Adjuvant chemotherapy			0.085
Yes	12(48.0)	77(31.0)	
No	13(52.0)	171(69.0)	
Local recurrence			0.068
Yes	2(8.0)	3(1.2)	
No	23(92.0)	245(98.8)	
Metastasis			0.441
Yes	1(4.0)	5(2.0)	
No	24(96.0)	243(98.0)	
Death			0.148
Yes	3(12.0)	12(4.8)	
No	22(88.0)	236(95.2)	

CEA: carcinoembryonic antigen, CA19-9: carbohydrate antigen 199, ECOG: Eastern Cooperative Oncology Group, pT stage: Pathological T stage, LNs: Lymph nodes

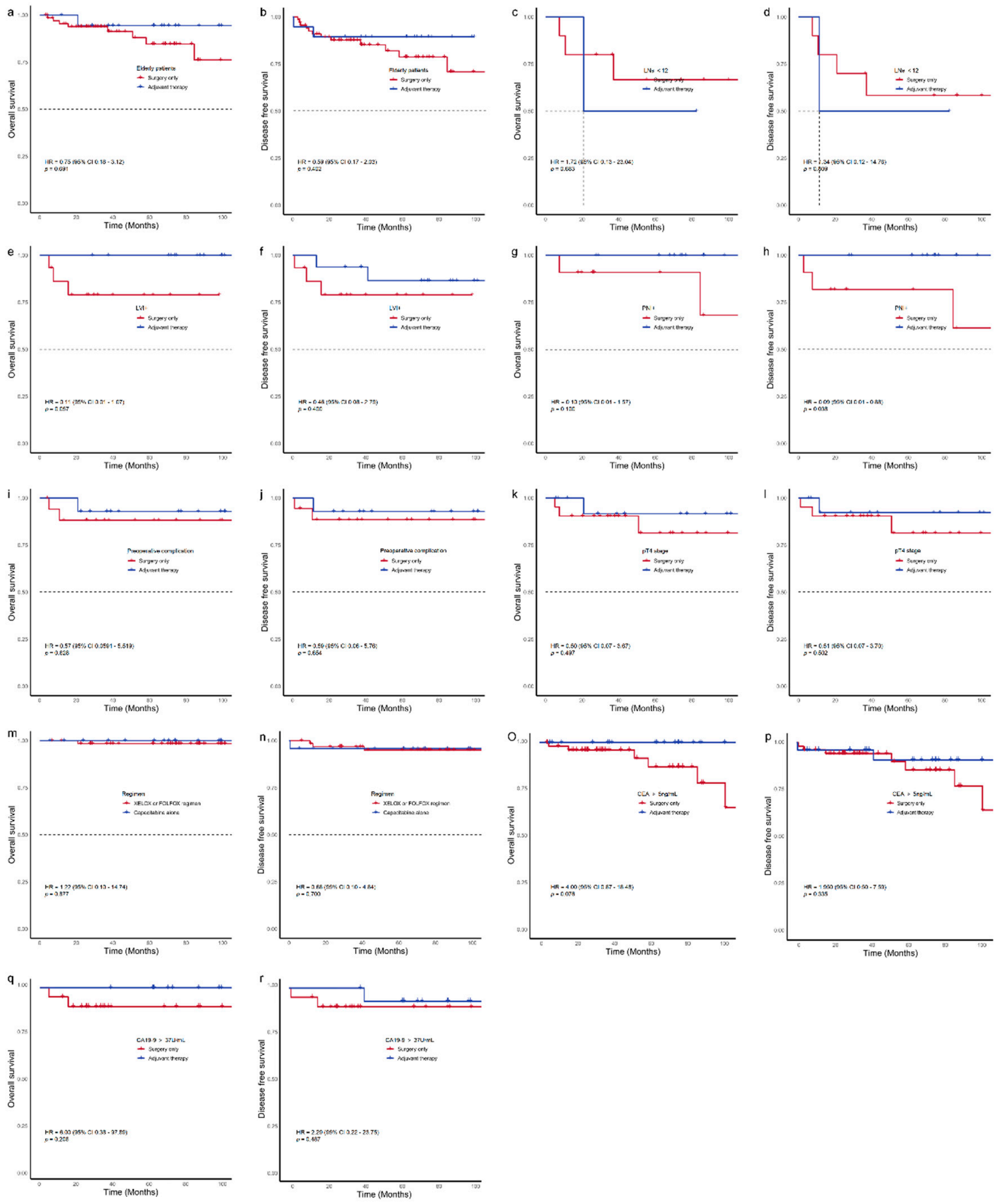

Supplement Figure S1 Kaplan–Meier survival curves for overall-survival (OS) and disease-free survival (DFS) of patients with stage II dMMR colon cancers stratified by other variables and adjuvant therapy regimens. (a) OS in elderly patients group. (b) DFS in elderly patients group. (c) OS in fewer than 12 examined lymph nodes (LNs ＜ 12) group. (d) DFS in LNs ＜ 12 group. (e) OS in lymphovascular invasion positive (LVI+) group. (f) DFS in LVI+ group. (g) OS in perineural invasion positive (PNI+) group. (h) DFS in PNI+ group. (i) OS in preoperative complication group. (j) DFS in preoperative complication group. (k) OS in pT4 stage group. (l) DFS in pT4 stage group. (m) OS in XELOX or FOLFOX regimen group and Capecitabine alone group. (n)

DFS in XELOX or FOLFOX regimen group and Capecitabine alone group. (o) OS in CEA > 5ng/mL group. (p) DFS in CEA > 5ng/mL group. (q) OS in CA19-9 > 37U/mL group. (r) DFS in CA19-9 > 37U/mL group.

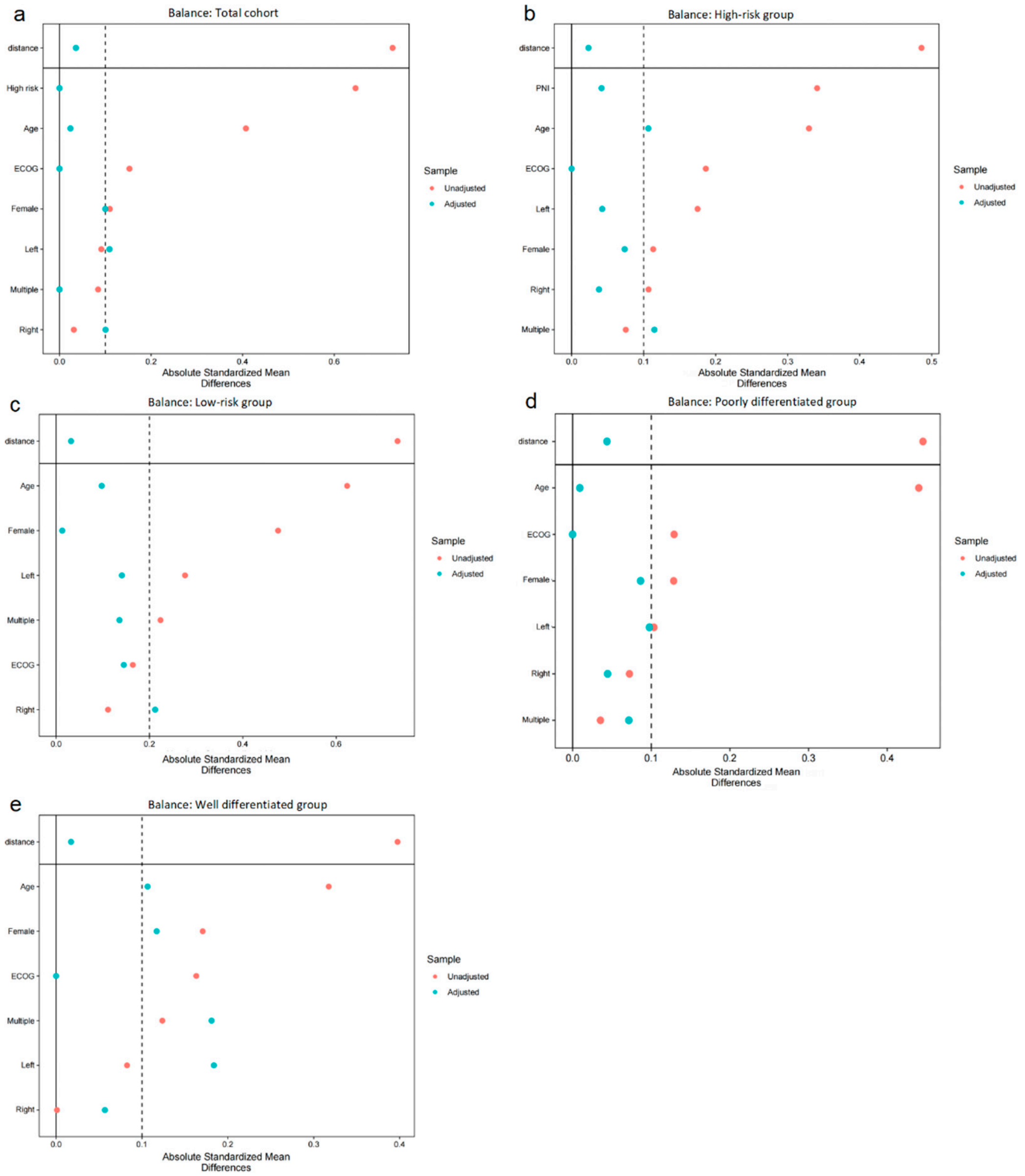

Supplement Figure S2 Covariate balance before and after adjustment in the: (a) total cohort; (b) high-risk group; (c) low-risk group; (d) poorly differentiated group; (d) well differentiated group.
